# A C-shaped miniaturized coil for transcranial magnetic stimulation in rodents

**DOI:** 10.1088/1741-2552/acc097

**Published:** 2023-03-24

**Authors:** Wenxuan Jiang, Robert Isenhart, Charles Y Liu, Dong Song

**Affiliations:** 1 Department of Biomedical Engineering, University of Southern California, Los Angeles, CA, United States of America; 2 Rancho Los Amigos National Rehabilitation Center, Downey, CA, United States of America; 3 USC Neurorestoration Center, University of Southern California, Los Angeles, CA, United States of America; 4 Department of Neurological Surgery, University of Southern California, Los Angeles, CA, United States of America

**Keywords:** TMS, rodent models, coil, electrophysiology, SSEP, MEP

## Abstract

*Objective.* Transcranial magnetic stimulation (TMS) is a non-invasive technique widely used for neuromodulation. Animal models are essential for investigating the underlying mechanisms of TMS. However, the lack of miniaturized coils hinders the TMS studies in small animals, since most commercial coils are designed for humans and thus incapable of focal stimulation in small animals. Furthermore, it is difficult to perform electrophysiological recordings at the TMS focal point using conventional coils. *Approach.* We designed, fabricated, and tested a novel miniaturized TMS coil (4-by-7 mm) that consisted of a C-shaped iron powder core and insulated copper wires (30 turns). The resulting magnetic and electric fields were characterized with experimental measurements and finite element modeling. The efficacy of this coil in neuromodulation was validated with electrophysiological recordings of single-unit activities (SUAs), somatosensory evoked potentials (SSEPs), and motor evoked potentials (MEPs) in rats (*n* = 32) following repetitive TMS (rTMS; 3 min, 10 Hz). *Main results.* This coil could generate a maximum magnetic field of 460 mT and an electric field of 7.2 V m^−1^ in the rat brain according to our simulations. With subthreshold rTMS focally delivered over the sensorimotor cortex, mean firing rates of primary somatosensory and motor cortical neurons significantly increased (154 $ \pm $ 5% and 160 $ \pm $ 9% from the baseline level, respectively); MEP and SSEP amplitude significantly increased (136 $ \pm $ 9%) and decreased (74 $ \pm $ 4%), respectively. *Significance.* This miniaturized C-shaped coil enabled focal TMS and concurrent electrophysiological recording/stimulation at the TMS focal point. It provided a useful tool to investigate the neural responses and underlying mechanisms of TMS in small animal models. Using this paradigm, we for the first time observed distinct modulatory effects on SUAs, SSEPs, and MEPs with the same rTMS protocol in anesthetized rats. These results suggested that multiple neurobiological mechanisms in the sensorimotor pathways were differentially modulated by rTMS.

## 1. Introduction

Transcranial magnetic stimulation (TMS) is a non-invasive neuromodulation technique extensively used in clinical applications and basic research. During TMS, a coil is placed over the scalp. A brief high-intensity current pulse passes through the coil producing a time-varying magnetic field (*B*-field). The *B*-field further induces an electric field (*E*-field) inside the brain, which modulates brain activities [1, 2]. Despite the widespread use of TMS for treating neurological and neuropsychiatric conditions such as depression [3–6], obsessive-compulsive disorder [7, 8], migraine [9], stroke [10], epilepsy [11], schizophrenia [12], and autism [13], the underlying mechanisms of its effects remain largely unclear. Animal models are essential for investigating such underlying mechanisms since they allow more comprehensive electrophysiological recordings and invasive experiments that are difficult or impractical on humans. However, most commercial TMS coils are designed for humans. The large geometric size of human TMS coils will cause non-focal high-intensity stimulation, which are not applicable to small animal studies [14–16]. Therefore, there is a strong need for miniaturized TMS coils in neuroscience, neural engineering, and clinical research communities.

**Figure 1.**
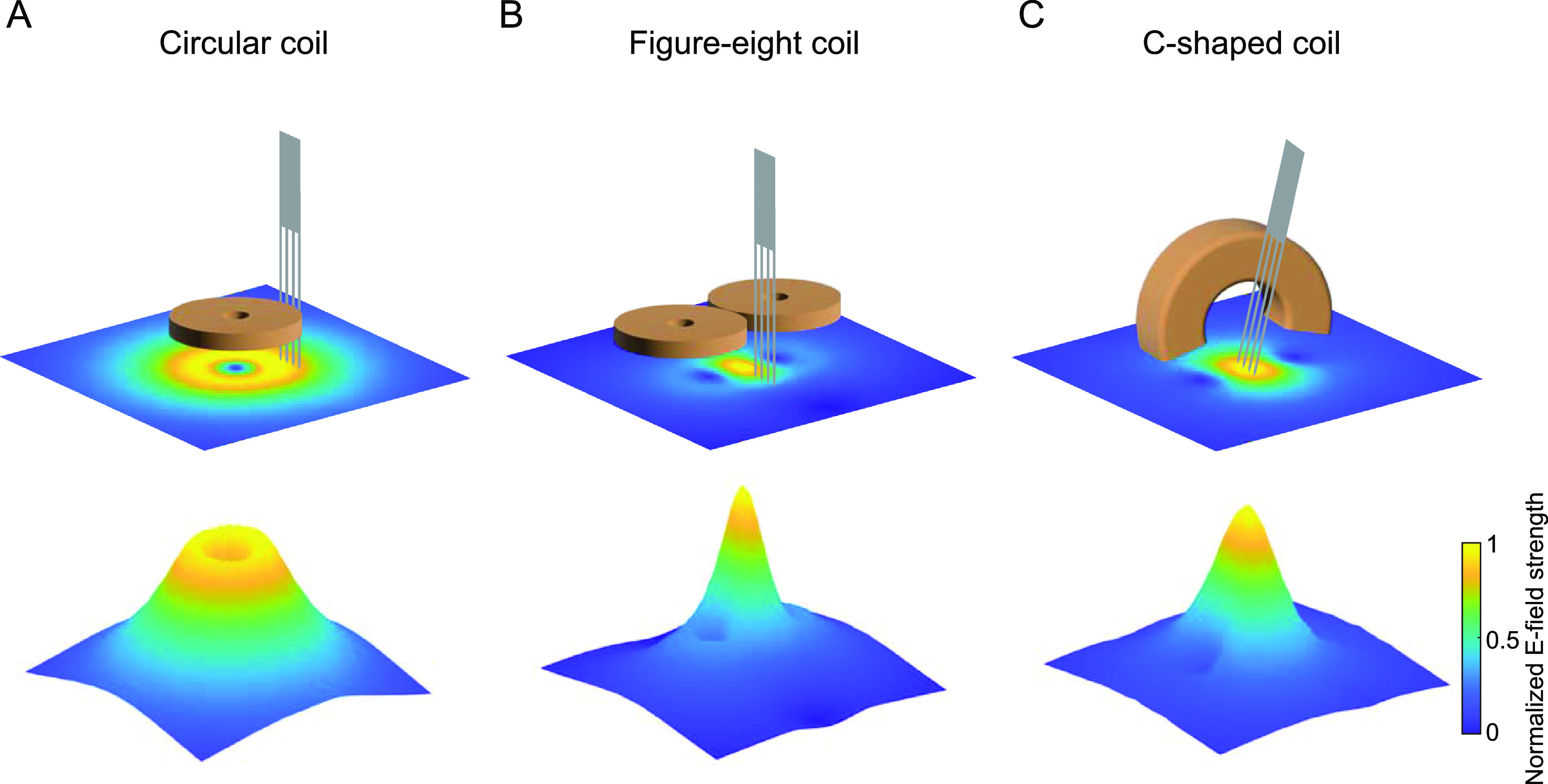
*E*-fields simulated with different TMS coils in free space. (A) A circular coil generated a non-focal maximum *E*-field under its perimeter. Standard multi-electrode array (MEA) could be implanted to the location with high *E*-field without obstruction. (B) A figure-eight coil generated a focal maximum *E*-field under the center of the coil. However, standard MEA could not be vertically implanted under the center of the coil unless the coil was far apart or tilted at a very large angle, which might result in weak or different *E*-field distribution. (C) A C-shaped coil generated a focal maximum *E*-field under the center of the coil. Standard MEA could be implanted to the focal point with the coil or the MEA slightly tilted.

Several miniaturized TMS coils have been developed for rodent studies, among which circular coil is one of the most popular designs. For example, a circular coil (8 mm outer diameter) with 300 turns of copper wire around an iron core was built to achieve a maximum *B*-field of 12 mT [17, 18]. By increasing the windings to 780 turns, it produced a maximum *B*-field of 120 mT and an *E*-field of 12.7 V m^−1^ on the brain surface at ∼1 mm below the coil [19]. A similar circular coil (9.2 mm outer diameter) with 70 turns of copper wire around a soft ferrite core produced a maximum *B*-field of 180 mT and an *E*-field of 2.5 V m^−1^ at 2 mm below the coil [20]. In another circular coil (5 mm outer diameter) with 50 turns of copper wire around a tapered iron powder core [21], a maximum *B*-field of 685 mT and an *E*-field of 15 V m^−1^ at the base of the coil were generated. However, the maximum *E*-field of circular coils has a circular shape under the coil perimeter thus is not focal (figure 1(A)) [22, 23]. In several recent studies, figure-eight coils were built for focal TMS in rodents. For example, a figure-eight coil with 9 turns of copper wire in each wing (19 mm outer diameter) could produce more focal *B*-field (300 mT as maximum) and *E*-field (0.55 V m^−1^ as maximum) [24]. A more powerful figure-eight coil (25 mm outer diameter) was designed to use a commercial stimulator to generate *B*-field and *E*-field intensities similar to those of human TMS coils that could elicit unilateral motor evoked potentials (MEPs) in rats, which was not achieved in most rodent studies [25]. With the aid of convex optimization for TMS in realistic rat head models [26, 27], the figure-eight coil could be further optimized with windings extending to a wide region (116 mm by 84 mm) to achieve minimum energy without sacrificing the stimulation focality [28]. To date, figure-eight coils in conjunction with specially modified flat electrodes or probes have been used to assess brain circuitry and function [29, 30]. However, the figure-eight coil cannot be easily combined with standard electrophysiology since it is difficult to implant a straight penetrating electrode under the center of the coil (where maximum stimulation intensity exists) while keeping the coil close to the brain surface (figure 1(B)). Other alternative designs [31–34] improved the stimulation focality using analytical models but at the same time generated *E*-field distributions different from those of circular or figure-eight coils, which are most commonly used in humans. Consequently, the neural responses elicited with these coils cannot be directly compared with those in human TMS studies.

To develop a TMS coil that can (1) generate focal *B*- and *E*-fields similar to those of human figure-eight coils, and (2) is compatible with standard electrophysiological recording and stimulation, we designed, fabricated, characterized, and evaluated a novel C-shaped miniaturized coil for use in small animals [35, 36] (figure 1(C)). This coil consisted of insulated copper wires around a C-shaped iron powder core, which allowed convenient implantation of electrophysiological recording and stimulation electrodes between the two bases of the coil. The resulting *B*- and *E*-fields were simulated with finite element modeling and further characterized with experimental measurements. The efficacy of this TMS coil in neuromodulation was validated with electrophysiological recordings of single-unit activities (SUAs), somatosensory evoked potentials (SSEPs), and MEPs before and after repetitive TMS (rTMS) in rats using single electrodes and multi-electrode arrays. This novel miniaturized coil and the associated experimental paradigm enabled the combination of TMS, electrophysiological recording, and electrical stimulation in rat brains. Using this paradigm, we for the first time observed distinct modulatory effects on SUAs, SSEPs, and MEPs with the same rTMS protocol in anesthetized rats. It provided a useful tool to investigate the neural responses as well as the underlying neurobiological mechanisms of TMS in small animal models.

## 2. Method

### 2.1. TMS coil design

A miniaturized TMS coil was built by winding 30 turns of insulated 23-gauge copper wire on a C-shaped magnetic core. The core was made from an iron powder toroid (T60-26, Micrometals, Anaheim CA, USA). The toroid has an outer diameter of 15.2 mm, an inner diameter of 8.5 mm, and a height of 6.0 mm. A small gap was cut on the toroid to form the C-shaped geometry. The angle and shortest distance between the two bases of the C-shaped coil were 150° and 5 mm, respectively. The winding at each base of the coil was rectangular with a dimension of 7 mm × 4 mm. The core material was composed of ferromagnetic particles coated with organic compounds to ensure electrical insulation. This insulation makes the core tolerant of thermal aging and provides high saturation flux density with low loss [21, 37, 38]. The permeability of the core material is 75 times that of the air, which greatly increases the magnetic flux density of the coil. The TMS coil was dip-coated with conformal silicone coating (422C, MG Chemicals, Canada) to provide extra protection and insulation. To characterize the electrical properties of the coil, the coil resistance and inductance were measured via a precision LCR meter (Keysight E4980AL, Santa Rosa, CA, USA) at 1 V input voltage from 20 Hz to 100 kHz (201 points in log scale).

### 2.2. TMS circuit design

**Figure 2.**
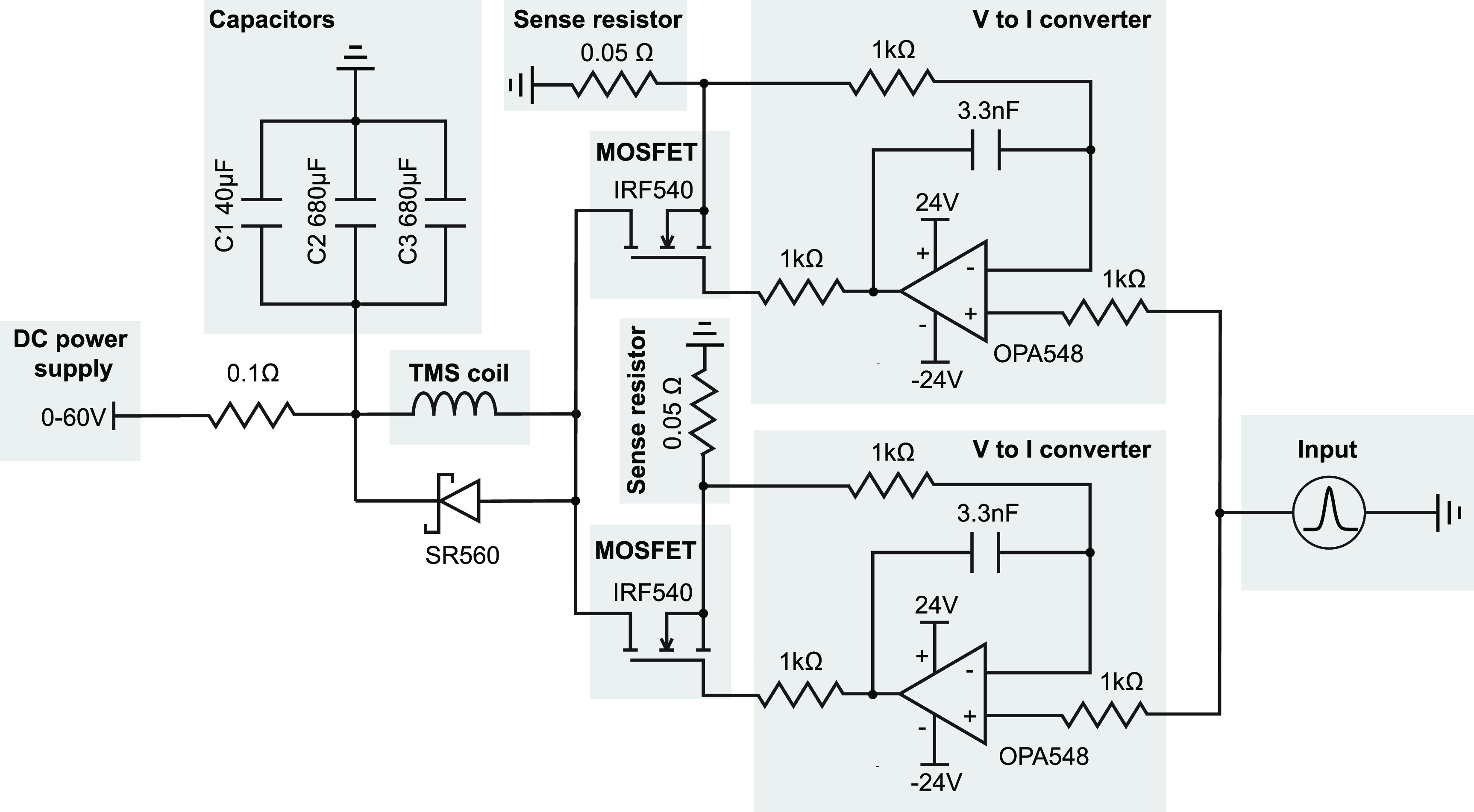
Simplified schematic of the driving circuit of the TMS coil. The stimulation pattern was generated with a waveform generator as the input to the circuit. The voltage signal was converted into current with two operational amplifiers. The output voltages controlled the on and off conditions of two MOSFETs in parallel. When the MOSFETs were off, the capacitors were charged up to the voltage of the DC power supply. When the MOSFETs were on, the capacitors were discharged, and a current pulse was generated in the TMS coil.

A custom-made stimulator was designed to generate TMS pulses through the coil (figure 2). A DC voltage source (Kungber SPS605 Variable DC Power Supply, Shenzhen, China) was used as the power supply to drive the circuit. Its voltage could be adjusted to control the amplitude of the TMS pulse. The maximal output of the DC voltage source (60 V) was used for all experiments. Two aluminum electrolytic capacitors (capacitance: 680 $\mu $F; voltage: 200 VDC) and one polymer film capacitor (capacitance: 40 $\mu $F; voltage rating: 500 VDC) were connected in parallel for generating the pulses. Since electrolytic capacitors had larger capacitance and resistance compared to film capacitor, the combination of those capacitors improved performance by providing high capacitance at low frequencies while providing low resistance at high frequencies. Two N-channel metal–oxide–semiconductor field-effect transistors (MOSFETs; IRF540, Vishay, Malvern PA, USA) were used to control the charging and discharging of the capacitors. When the voltage applied at the gate terminal was below the threshold voltage, the MOSFET was off, and the capacitors was charged up to the supplied DC voltage. When the voltage applied at the gate terminal was above the threshold voltage, the flow of current through the MOSFET led to discharging of capacitors. As a result, a current pulse was generated in the TMS coil. A Schottky diode (SR560, EIC semiconductor, Bangkok, Thailand) parallelly connected to the coil was used to protect the MOSFETs from damage. The gate voltage of the MOSFET was determined by the output of an operational amplifier (OPA548, Texas Instruments, Dallas TX, USA). The noninverting input terminal of the amplifier was connected to an Agilent 33500B arbitrary waveform generator (Keysight, Santa Rosa, CA, USA), which could be programmed to generate monophasic waveforms as the stimulator’s input. The inverting input terminal of the amplifier was connected in feedback to a sense resistor (0.05 Ω) and the source terminal of the MOSFET. The high forward gain and differential input nature of the amplifier made it a voltage-to-current converter that drove the gate of the MOSFET. When it was above the threshold, the voltage of the input signal appeared across the sense resistor. The current passing through the sense resistor would appear in the drain of the MOSFET and the TMS coil. Two identical MOSFET circuits were used and connected in parallel to double the coil current. Therefore, the coil current was measured as the sum of currents across two sense resistors in the circuit.

To evaluate the performance of the stimulator, Gaussian pulses with different peak amplitudes (0–10 V) but the same standard deviation (30 *µ*s) were generated as input signals to the circuit. The coil current was measured at each intensity in an incremental order with steps of 0.2 V. In addition, the coil current was measured when the Gaussian pulses were delivered at different frequencies (1, 5, 15, and 20 Hz) with the same peak amplitude of 7.5 V and standard deviation of 30 *µ*s. To further test the flexibility and real-time capability of the device, arbitrary stimulation patterns of Gaussian pulses with random peak amplitude (3–8 V) and frequency (1–20 Hz) were generated via real-time Standard Commands for Programmable Instruments (SCPI) commands sent to the arbitrary waveform generator. The coil current was recorded simultaneously.

### 2.3. Measurements of magnetic and electric fields

**Figure 3.**
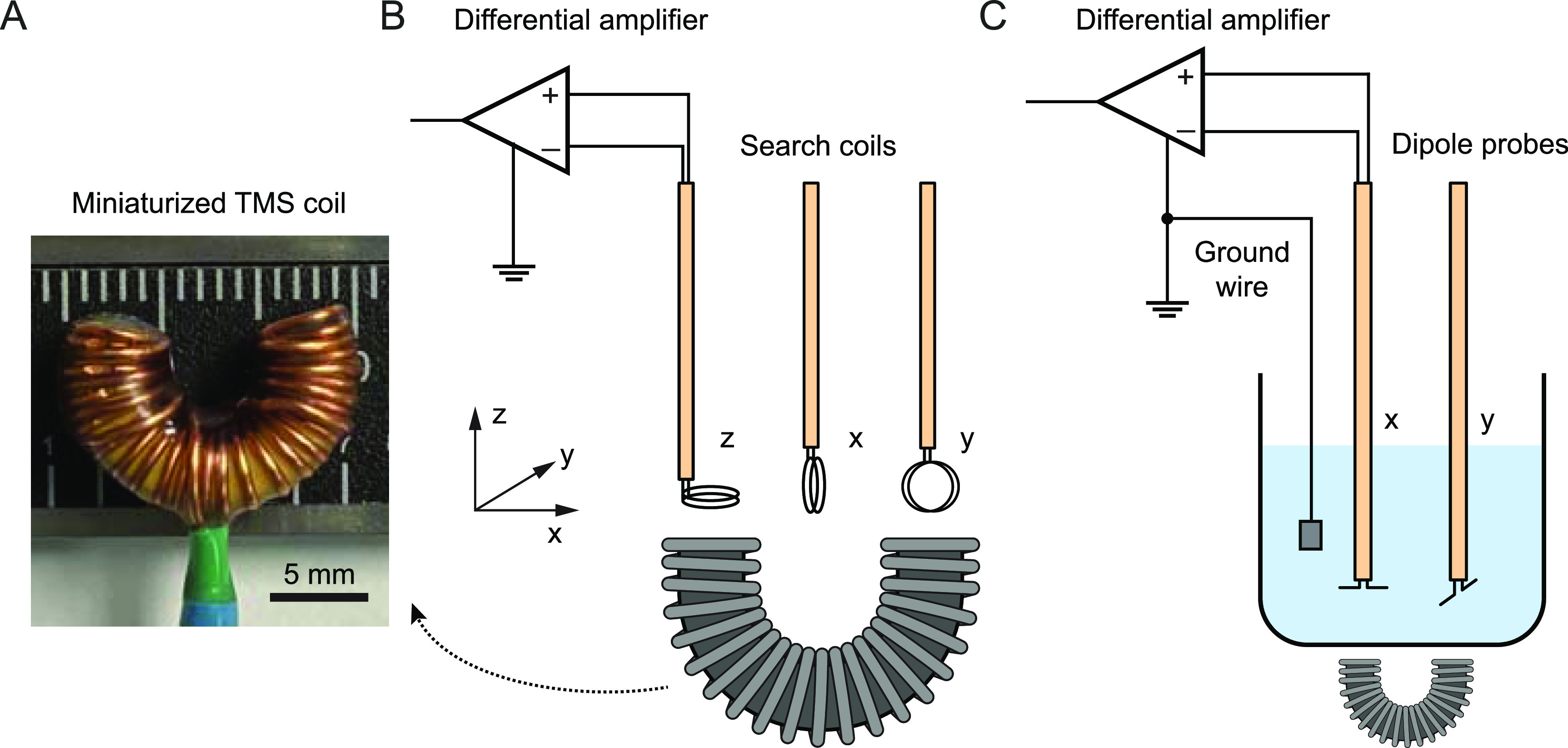
*B*- and *E*-field measurement. (A) Photograph of the miniaturized C-shaped TMS coil. (B) Schematic of the set-up for *B*-field measurement. Three types of search coils were placed above the coil to measure the *B*-field in *x-, y*- and *z*-directions. (C) Schematic of the set-up for induced *E*-field measurement. Two dipole probes were placed above the coil and oriented to measure the *E*-field in *x*- and *y*-directions within saline.

To characterize the C-shaped TMS coil (figure 3(A)) driven by the stimulator, experimental measurements of *B*- and *E*-fields were performed with a stereotaxic system in a Faraday cage. The center point between two bases of the coil was defined as the origin of the 3D coordinates. The *x-, y*-, and *z*-component of *B*-field (${B_x},\,{B_y},\,{B_z}$) produced by the TMS coil were measured using a two-loop search coil (radius *r*= 1.15 mm, number of turns *N*= 2) (figure 3(B)). The search coil was placed above the base of the TMS coil and oriented to the direction of each measured component. Voltage *V* was induced in the search coil during stimulation and amplified 100 times with a Model 1700 Differential AC Amplifier (A-M systems, Sequim WA, USA). The *B*-field was sampled with a 1 mm spatial resolution. The induced voltages along each direction (${V_x},\,{V_y},\,{V_z}$) in the transverse (*x*–*y*) plane were captured with an Axon Digidata 1322 A acquisition system (Molecular Devices, Sunnyvale CA, USA). Assuming the *B*-field was homogeneous over the search coil, Faraday’s law of induction was used to calculate each component of *B*-field [39]:

\begin{equation*}{B_{x,y,z}} = - \frac{1}{{N{r^2}\pi }}{\mathop \int \nolimits }{V_{x,y,z}} \cdot {\text{d}}t\end{equation*}

The corresponding *x*- and *y*-component of *E*-field (${E_x},\,{E_y}$) were measured using a dipole probe in saline (figure 3(C)). The dipole probe consisted of two twisted insulated wires with a 3 mm separation at the exposed ends [40, 41]. The TMS coil was placed underneath a glass beaker that was filled with sodium chloride solution (0.9%). The dipole probe was placed in the solution and oriented to the direction of each component. A biphasic waveform was measured via the dipole probe during stimulation. It was amplified 1000 times with a DAM50 differential amplifier (World Precision instruments, Sarasota FL, USA). The *E*-field was sampled with a 2 mm spatial resolution. The induced voltages along each direction in the transverse plane were measured with the Axon data acquisition system. Assuming the induced *E*-field was approximately constant between the exposed ends of the twisted wires [40], the *x*- and *y*-components of *E*-field were calculated as:

\begin{equation*}{E_{x,y}} = \, - \frac{{{{\Delta }}V_{x,y}}}{{\Delta l_{x,y}}}\end{equation*}

where ${{\Delta }}V_{x,y}$ was the first peak of the biphasic waveform measured along *x*- and *y*-direction, respectively; $\Delta l_{x,y}$ was the known distance of the exposed ends along each direction.

### 2.4. Finite element modeling of magnetic and electric fields

Finite element modeling was conducted using Multiphysics 6.0 (COMSOL, Burlington MA, USA) to simulate the *B*- and *E*-fields induced by the TMS coil. The magnetic core was constructed as a half circle (−105° to 105°) with a rectangular transection (6 mm × 3 mm). Relative permeability of the core material was set to 75; the electrical conductivity was set to 0 due to the dielectric coating of iron powder; the winding was simulated as a single copper conductor with a diameter of 0.2 mm. The 30-turn helical coil was evenly distributed on the core. In the frequency domain analysis, the coil current, ${I_{{\text{coil}}}}$, was modeled as a sinusoidal current at frequency, ${f_{{\text{sine}}}}$, with the same peak amplitude, ${I_{\max }}$, and the same maximum rate of change at time, $t = {t_0}$:

\begin{equation*}\frac{{{\text{d}}{I_{{\text{coil}}}}}}{{{\text{d}}t}}{|_{t = {t_0}}} = 2\pi {f_{{\text{sine}}}}{I_{\max }}\end{equation*}

As a result, the TMS coil was injected with a 142 A sinusoidal current at 3217 Hz to replicate the maximum time derivative of 2.87 A $\mu $s^−1^ in measured Gaussian current pulse.

For comparison with the experimental measurements, the *B*-field was simulated in the air; the *E*-field was simulated in saline. A cylinder with a height of 60 mm and a diameter of 60 mm was created to approximate the saline solution in the beaker. Its isotropic electrical conductivity was set at 1.45 S m^−1^ [42]. The simulated TMS coil was positioned underneath the cylinder as in the experimental setup. *B*- and *E*-fields were calculated using COMSOL Magnetic and Electric Fields (mef) interface. Complete meshes of *B*- and *E*-fields consisted of 204 3805 and 492 000 elements, respectively.

To estimate the *B*- and *E*-fields induced in the brain, a 3D rat brain model was built based on an existing model [43], which was constructed from 240 cross-sectional magnetic resonance imaging slices. Electrical conductivity of the brain was set to 0.106 S m^−1^ corresponding to gray matter [41, 44, 45]. The TMS coil was positioned 1.5 mm above the right hemisphere and rotated 15° as in the experimental setup. The complete mesh consisted of 188 150 elements.

### 2.5. Animal surgery

Female Sprague-Dawley rats (*n* = 32, 11–12 weeks, 220–250 g) were used in this study; eight animals were used for single-unit recordings from the primary somatosensory cortex (S1); eight animals were used for single-unit recordings from the primary motor cortex (M1); eight animals were used for SSEP recordings while another eight animals were used for MEP recordings.

Animals were anesthetized with ketamine-xylazine (K, 75 mg kg^−1^; X, 10 mg kg^−1^; intraperitoneal administration) following a rapid inhalational induction with 3%–4% isoflurane. Anesthesia was maintained with additional boluses of 36 mg kg^−1^ ketamine. The animal was mounted on a stereotaxic frame with Parafilm-wrapped ear bars and nose cone in a Faraday cage. Neural recordings were conducted when the anesthesia state was stable. Physiological status of animals (pedal reflex, body temperature, breathing rate, etc) was monitored throughout the experiments. A craniotomy was performed to expose the right sensorimotor cortex. One burr hole was incised to attach the ground wire over the occipital bone.

### 2.6. Single-unit recordings

**Figure 4.**
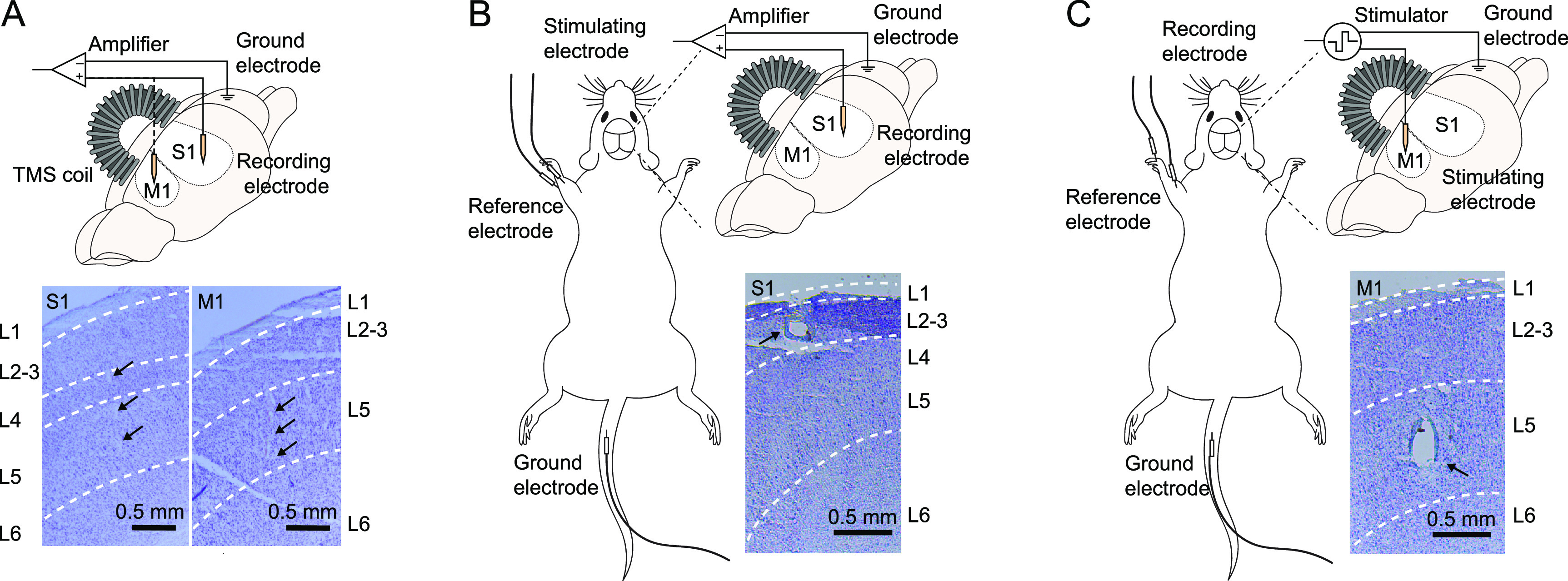
TMS combined with electrophysiological recording and stimulation. (A) Experimental set-up for single-unit recordings. Single-unit activities were recorded with the MEA implanted in the S1 and the M1 regions. The coil was placed above the sensorimotor cortex over the craniotomy window. The location of MEA placement was verified with an implantation lesion (S1 layer 4–5 and M1 layer 5). (B) Experimental set-up for SSEP recording. SSEPs elicited by electrical stimulation in the forelimb were recorded with the microelectrode implanted in the S1 region. Microelectrode location was verified with an electrolytic lesion (S1 layer 2–3). (C) Experimental set-up for MEP recording. MEPs elicited by electrical stimulation in the M1 were recorded with needle electrodes inserted in the forelimb. Stimulating microelectrode location was verified with an electrolytic lesion (M1 layer 5).

To conduct single-unit recordings, a multi-electrode array (Neuronexus A8x8-Edge-5 mm-50-150-177, Ann Arbor MI, USA) was implanted in the S1 (AP: 0.00 mm; ML: 3.80 mm) or the M1 (AP: 2.40 mm; ML: 3.00 mm) regions (figure 4(A)). SUAs were recorded and sorted via OmniPlex neural recording system (Plexon, Dallas TX, USA).

By connecting the recording system with implanted electrodes, wire loops between electrodes were formed [30, 41]. During TMS, the changing magnetic field would induce current in the loop. The injected current via electrodes might evoke neural responses at high amplitude. To estimate the amount of TMS-induced current in the recording electrode, the induced electromotive force around the wire loop formed with the recording assembly and coupled to the maximal *B*-field during TMS without any offset was calculated [41]:

\begin{align*}{\text{max}}\left| \varepsilon \right| &amp; = {\text{max}}\left| {\frac{{\text{d}{\phi _B}}}{{\text{d}t}}} \right| = \left| {\frac{{{e^{ - \frac{1}{2}}}{B_{\text{max}}}S}}{\sigma }} \right| \nonumber\\ &amp;= \,\frac{{{e^{ - \frac{1}{2}}} \times 473\,{\text{mT}} \times \,500\,{\text{m}}{{\text{m}}^2}}}{{30\,\mu {\text{s}}}} = 4.8\,{\text{V}}\end{align*}

where $\varepsilon $ is the induced electromotive force; ${\phi _B}$ is the magnetic flux through the loop; ${B_{\max }}$ is the maximal *B*-field during TMS; *S* is the area of the loop formed by the recording electrode and reference electrode; $\sigma $ is the standard deviation of the Gaussian-shaped pulse. The induced current in the loop could be converted using the input impedance of the headstage (40 M${{\Omega }}$) yielding 120 nA. It is unlikely to induce significant neural responses via microsimulation at this amplitude [46, 47], and the induced current could be even smaller due to the offset of the wire loop. Similarly, the induced current in the ground electrode could be converted using the impedance of the rat body with a larger loop area. However, the injected current density via ground electrode could be negligible due to a large contact area.

### 2.7. Somatosensory evoked potentials (SSEPs)

To record SSEPs, a single platinum–iridium microelectrode (PI2PT30.5A5, Microprobes, Gaithersburg MD, USA) was implanted in the S1 (AP: −0.25 mm; ML: 3.80 mm) (figure 4(B)). The microelectrode and ground wire were connected to the DAM50 differential amplifier (gain: 1000). Two subdermal needle electrodes (Natus, Middleton WI, USA) were inserted into the contralateral forelimb to stimulate the median nerve [48]. Another needle electrode was inserted into the tail as the ground for a STG1004 stimulator (Multi Channel Systems, Reutlingen BW, Germany). A biphasic square current pulse (pulse amplitude: ±0.8 mA; pulse width: 0.1 ms) was applied every 5 s to evoke SSEPs. All signals were recorded with the Axon data acquisition system.

### 2.8. Motor evoked potentials (MEPs)

To record MEPs, a single microelectrode was implanted in the M1 (AP: 1.25 mm; ML: 3.00 mm) (figure 4(C)). The microelectrode and ground wire were connected to the STG1004 stimulator. Two biphasic current pulses (pulse amplitude: ±0.8 mA; pulse width: 0.5 ms; inter-pulse interval: 2 ms) were applied every 5 s to evoke MEPs. To record muscle activities, two subdermal needle electrodes were inserted into the biceps brachii muscle and the finger pad of the contralateral forelimb as the recording and reference electrode, respectively [30, 48]. A third needle electrode was inserted to the tail as the ground. MEPs were recorded with the DAM50 differential amplifier (gain: 1000) and Axon data acquisition system.

### 2.9. Repetitive transcranial magnetic stimulation (rTMS)

rTMS in all animal experiments were delivered as Gaussian input pulses with a peak amplitude of 7.5 V and standard deviation of 30 *µ*s at 10 Hz. During single-unit or evoked potential recordings, the TMS coil was placed ∼1.5 mm above the cortex. It was rotated 15° to make the tip of the electrode align with the coil center. Following 5 min baseline recordings, subthreshold rTMS (3 min, 10 Hz) or control condition (rTMS turned off) was delivered to the sensorimotor cortex (*n* = 5, rTMS groups; *n* = 3, control groups). To evaluate the time-course of rTMS effects, neural signals were monitored for 15 min after stimulation.

### 2.10. Temperature measurement

To evaluate the heating effects produced by the coil, the temperature of the coil and the brain surface before and after rTMS (3 min, 10 Hz) was measured three times using a thermometer (Famidoc, Guangdong, China) in three animals. The temperature of the coil was measured to be 23.8 $ \pm \;0.1$ °C before rTMS (10 Hz) and increased to 46.7 $\pm$ 0.3 °C after 3 min of rTMS (*n* = 3). Meanwhile, the temperature of rat brain surface was measured to be 35.8 $ \pm $ 0.2 °C before rTMS and slightly increased to 36.2 $ \pm $0.2 °C after 3 min of rTMS (*n* = 3).

### 2.11. Histology

Histology was performed to verify the implantation site in the brain. For evoked potential recordings, an electrolytic lesion was created by delivering a constant current (amplitude: 0.3 mA; duration: 10 s) to the implanted microelectrode. For single-unit recordings, no electrolytic lesions were created since the multi-electrode array could not be used to deliver a strong enough current. Perfusion was performed using 10% formalin solution (Sigma-Aldrich, Burlington MA, USA) via the vascular system. After the brain was extracted and dehydrated with 18% sucrose solution, brain slices (50 *μ*m) were cut on a coronal plane with a Cryostat (Leica, Buffalo Grove IL, USA). Nissl staining was applied on the brain slices to visualize the neuron populations and implantation lesions.

### 2.12. Data processing and statistical analyses

All neural recordings were imported and processed in MATLAB (MathWorks, Natick MA, USA). The baseline of the neural recordings was defined as the 5 min recording when the animal was under a stable anesthesia state prior to rTMS. For single-unit recordings, a 250 Hz highpass filter was first applied to the data. Spike sorting was performed in the Plexon offline sorter (Plexon, Dallas TX, USA) to extract the timestamps of neuronal firing. The mean firing rate of each neuron was calculated and averaged with a one-minute bin size. For evoked potential recordings, timestamps of current stimulus artifacts were captured by setting a threshold using MATLAB function ‘findpeaks’. The DAM50 amplifier was set with a bandpass filter of 0.1 Hz–10 kHz, and no other filter was applied to the evoked potentials. After the timestamps of each stimulus artifact were obtained, a 100 ms window following the artifact was extracted to isolate the evoked potentials. The individual peak-to-peak amplitude was calculated as the difference between the highest and the lowest value in each evoked potential. The individual latency was calculated as the duration between the stimulus artifact and the first negative peak of each evoked potential. The peak-to-peak amplitude and latency of each evoked potential were further averaged every minute (12 consecutive sweeps).

All values were normalized with their corresponding 5 min baseline recordings and reported as the mean ± standard error of the mean (SEM). Two-tailed *t*-tests with a significance level of 0.01 were conducted to compare the normalized neural signals before and after rTMS or control condition.

## 3. Results

### 3.1. Characterization of TMS coil

**Figure 5.**
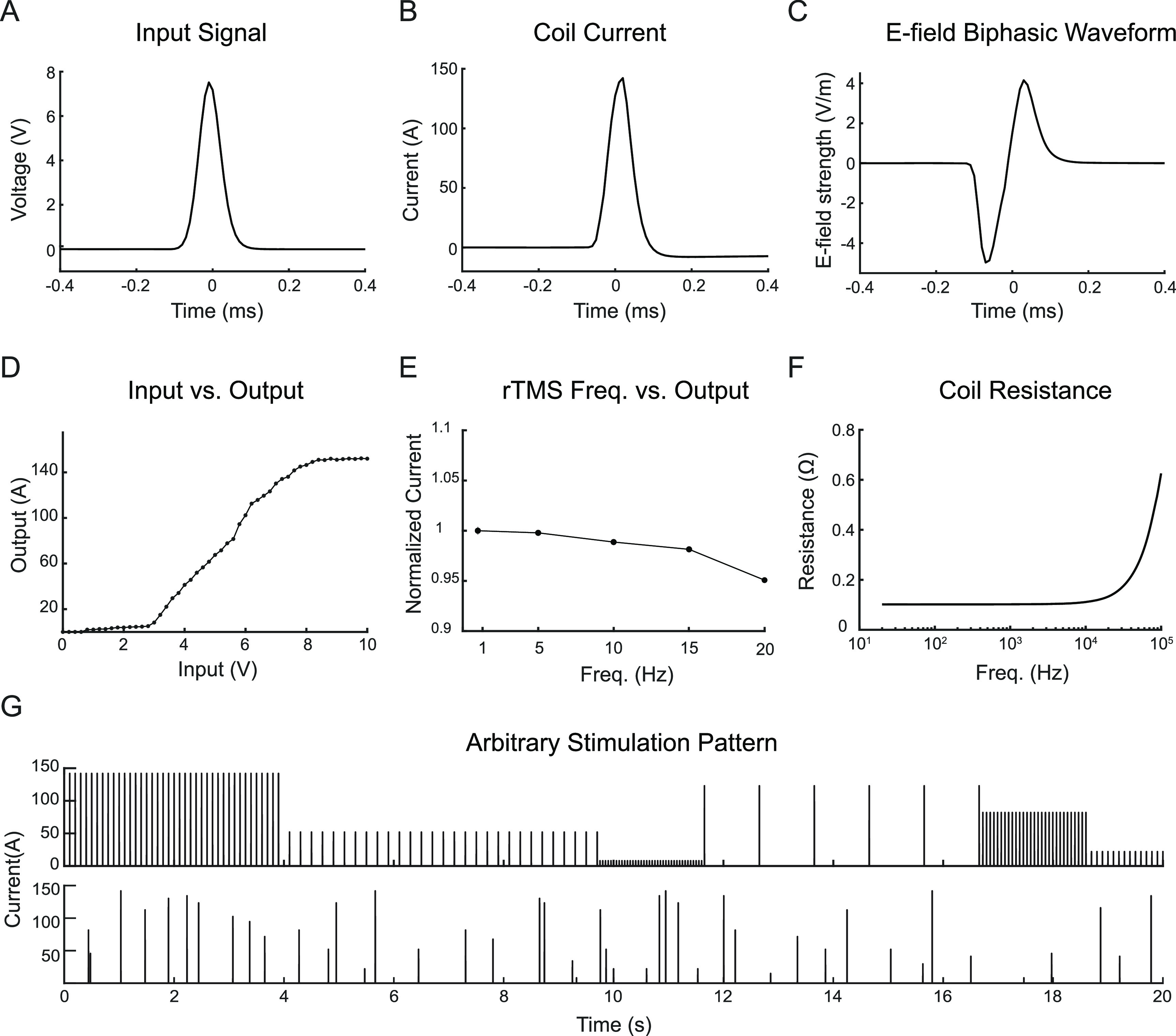
Electrical properties of the TMS coil. (A) A Gaussian voltage pulse generated by a waveform generator was used as the input signal. (B) Coil current. (C) *E*-field waveform measured in saline. (D) Output (coil) current as a function of input signals. (E) Output current relative to current measured at 1 Hz as a function of rTMS frequency. (F) Coil resistance measured via a precision LCR meter. (G) Representative arbitrary stimulation pulse patterns generated by the coil. Top: stimulation pattern with multi-level amplitudes and frequencies. Bottom: stimulation pattern with random amplitudes and intervals.

Gaussian pulses (peak amplitude: 7.5 V; standard deviation $\sigma $: 30 *µ*s) were generated as input signals to the circuit (figure 5(A)). The corresponding coil current was measured across the sense resistors (figure 5(B)). The current had the same shape as the input pulse and a peak amplitude (${I_{{\text{max}}}})$ at 142 A. The current as a function of time could be approximated by the following equation:

\begin{equation*}{I_{{\text{coil}}}}\left( t \right) = {I_{\max }}{e^{ - \frac{1}{2}{{\left( {\frac{t}{\sigma }} \right)}^2}}}\end{equation*}

The induced *E*-field waveform was proportional to the first derivative of the coil current. The resulting biphasic waveform was measured via a dipole probe in saline (figure 5(C)). The induced *E*-field waveform as a function of time could be approximated by the following equation:

\begin{equation*}E\left( t \right) = \frac{{{E_{\max }}}}{{{e^{ - \frac{1}{2}}}\sigma }}t \cdot {e^{ - \frac{1}{2}{{\left( {\frac{t}{\sigma }} \right)}^2}}}\end{equation*}

where ${E_{\max }}$ was measured to be 5.2 V m^−1^ when the dipole probe was placed ∼3.5 mm from the center of the coil. The input–output curve demonstrates the relationship between the input signal and the output (coil) current (figure 5(D)). The response of the circuit was linear when the input signal was within the range of 3–8 V. The lower limit (3 V) was determined by the gate-source threshold voltage of the MOSFET (2–4 V). However, saturation occurred when the input signal was greater than 8 V. In addition, the output current was affected by the rTMS frequency (figure 5(E)). The coil current decreased as the frequency increased. At 20 Hz, the coil current dropped to 95% of the value at 1 Hz. Furthermore, the resistance and inductance of the coil were measured via a precision LCR meter at 1 V input voltage from 20 Hz to 100 kHz. The resistance remained at 0.1 Ω when the frequency was lower than 10 kHz (figure 5(F)). This limit was much higher than the frequency of the input pulse (3217 Hz); therefore, the skin and proximity effects, which reduced the effective area of the copper wire and increased the overall resistance [49], were not obvious during stimulation. The inductance of the coil was measured to be constant at 11.7 *µ*H when the input frequency is between 100 Hz and 10 kHz, which was similar to the simulated inductance of 13.4 *µ*H in the finite element modeling. However, the inductance might vary with the amplitude of the coil current due to the use of the magnetic core which could introduce nonlinearity at high amplitude [50]. The TMS coil was able to deliver arbitrary stimulation patterns (figure 5(G)) via the custom-made stimulator. The pulse amplitude and frequency were precisely controlled through real-time SCPI commands sent to the arbitrary waveform generator.

### 3.2. Comparison of simulated and measured field distributions

**Figure 6.**
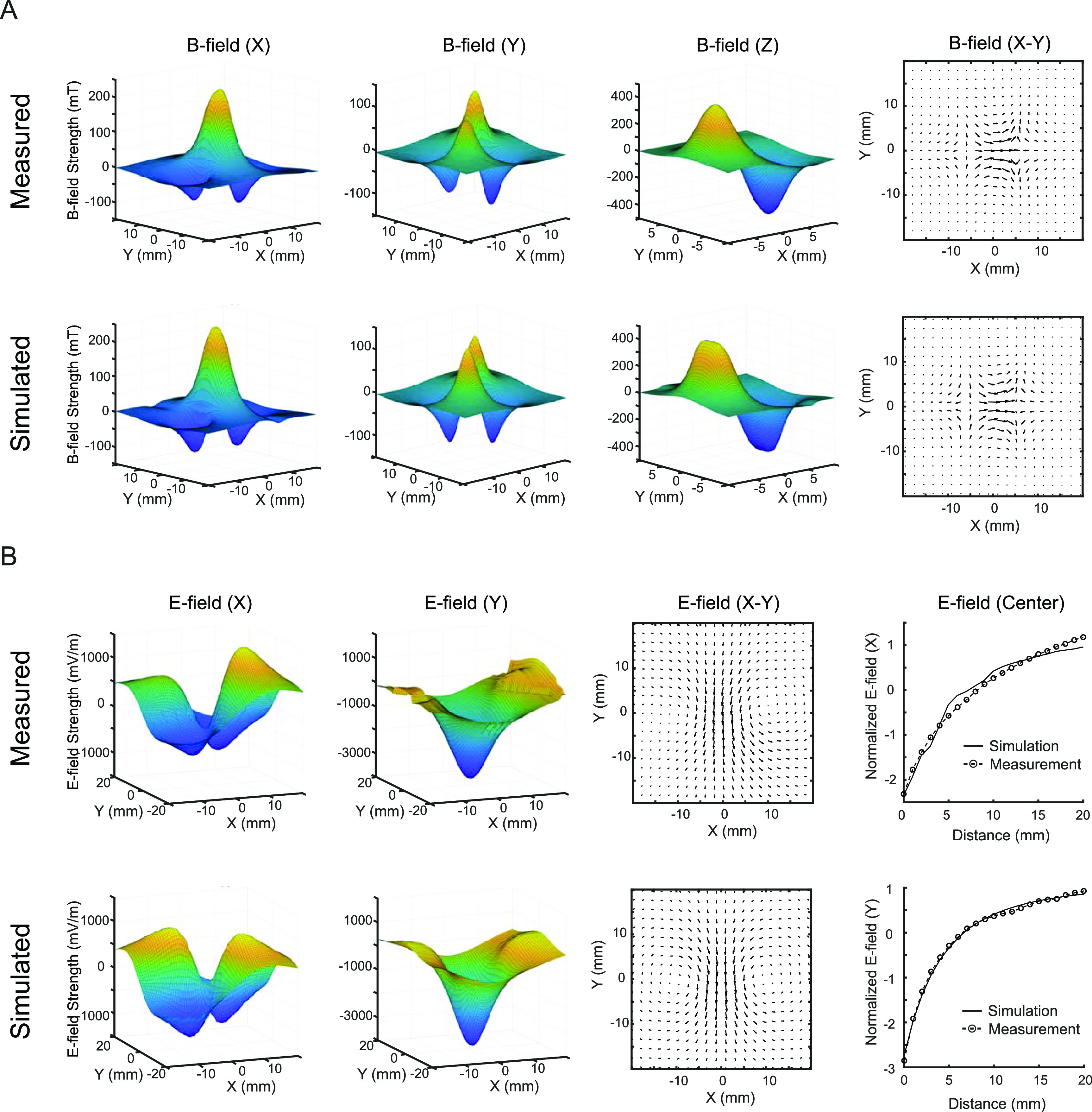
*B*- and *E*-field distributions from measurement and simulation. (A) Measured (top) and simulated (bottom) *B*-field profiles in the transverse plane. The *x-, y*-, and *z*-components of *B*-field and their combination of *x*- and *y*-components of *B*-field were in the air when the TMS coil was placed ∼0.5 mm away. (B) Measured (top) and simulated (bottom) *E*-field profiles in the transverse plane. The *x*- and *y*-components of *B*-field and their combination were in saline when the TMS coil was placed ∼4.5 mm away. In addition, normalized measured and simulated *x*-component (top) and *y*-component (bottom) of *E*-field as a function of depth along the *z*-direction showed consistent decay rates.

*B*-field induced by the coil in the air was measured on benchtop and simulated with finite element modeling method. The measured and simulated *B*-fields were highly consistent (figure 6(A)). At the center of the TMS coil, the peak strength of *x*-component of *B*-field was measured and simulated to be 227 mT and 247 mT, respectively. Both measurement and simulation showed two smaller peaks with opposite polarity at the bases of the coil. The measurement showed a slightly larger peak at the right base (south pole) compared with the left base (north pole). This deviance in magnitude might be due to the slight asymmetry of the handcrafted magnetic core. The *y*-component of *B*-field had four peaks with different polarity, which were apparent in both measurement and simulation. Among the four peaks, each pair of the diagonal peaks shared the same polarity. The combination of *x*- and *y*-component was shown in the quiver plots. The measurement and simulation showed near identical *B*-field distributions. The minimum points existed at the base of the coil. The main *B*-field vectors pointed from the north pole to the south pole. The *z*-component was the dominant component of *B*-field in the transverse plane. The simulation showed that the north pole and the south poles had peak strengths of 446 mT and −455 mT, respectively. The measurement showed that the north pole and south pole had peak strengths of 406 mT and −473 mT, respectively.

*E*-field induced by the coil in saline was further measured and simulated. The dipole probe was placed ∼4.5 mm from the base of the coil considering the glass thickness (∼3.5 mm). The measured and simulated *E*-fields were also highly consistent (figure 6(B)). The *x*-component of *E*-field had four peaks with different polarity in both measurement and simulation. Similar to the *y*-component of *B*-field, each pair of the diagonal peaks shared the same polarity, and each pair of the adjacent peaks had opposite polarity. At the center of the TMS coil, the peak strength of *y*-component of *E*-field was measured and simulated to be 3.7 V m^−1^ and 3.9 V m^−1^, respectively. Both measurement and simulation showed two smaller peaks with opposite polarity at the base of the coil. The combination of *x*- and *y*-component was shown in the quiver plots. The field vectors followed the direction of induced current produced by the changing *B*-field. The two ring-shaped fields under the two bases of the coil reinforced each other along *y*-direction and led to a stronger and more focal maximum *E*-field strength at the center. When the dipole probe was placed ∼3.5 mm from the center of the coil, a maximum strength of 5.2 V m^−1^ was achieved. To compare the measurement and simulation along *z*-direction, the dipole probe was moved vertically at the center of the coil. Results showed high degree of consistency in the decay rate for both *x*- and *y*-components.

### 3.3. Magnetic and electric field distribution in the rat brain

**Figure 7.**
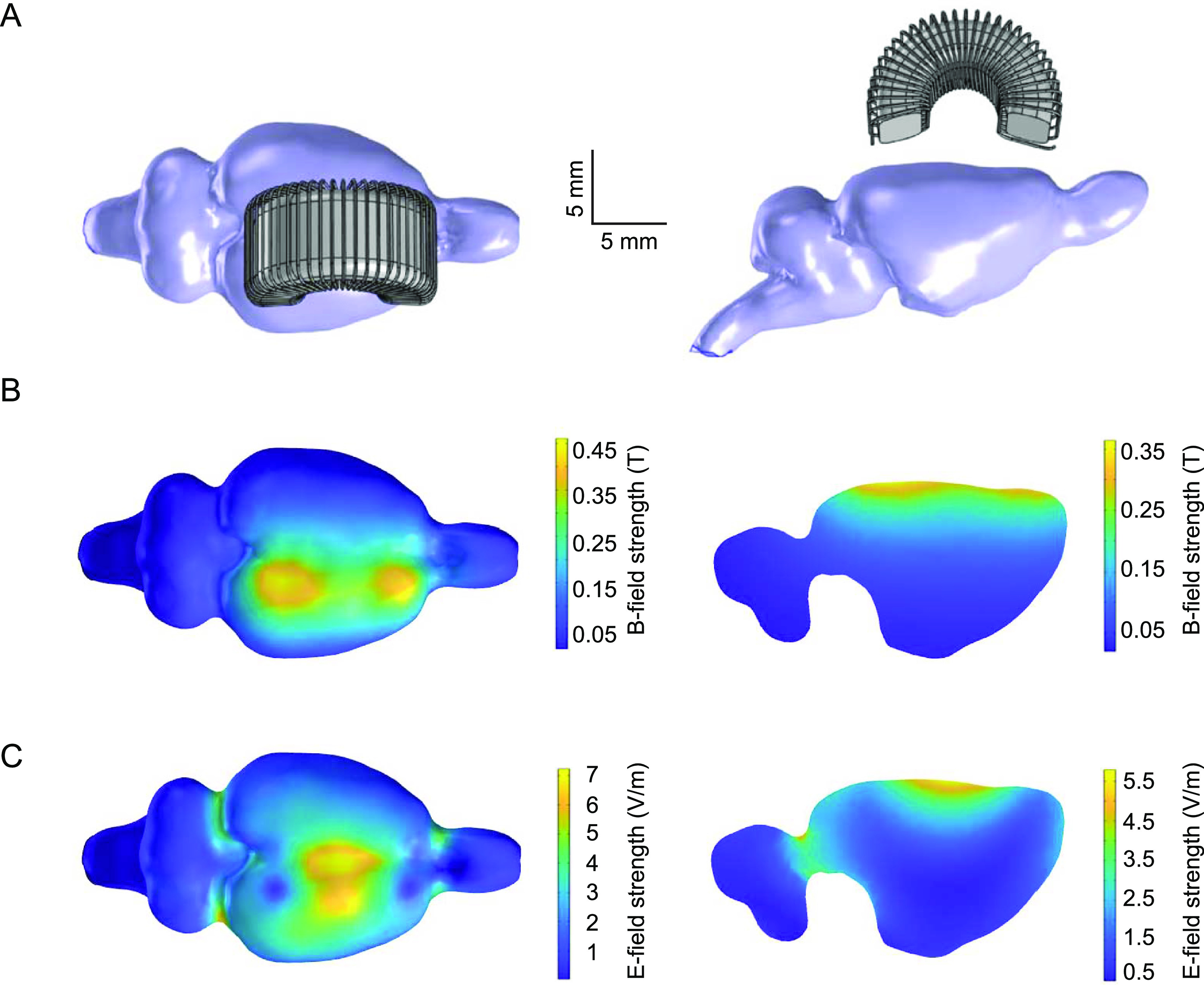
Simulated *B*- and *E*-field distributions in the rat brain. (A) Superior (left) and sagittal (right) views of coil placement relative to a 3D rat brain. (B) *B*-field distribution on the surface (left) and sagittal section (right) of the brain. (C) *E*-field distribution on the surface (left) and sagittal section (right) of the brain.

*B*- and *E*-fields in the rat brain were further simulated with the finite element modeling method (figure 7). In the simulation, the C-shaped coil was placed above the sensorimotor cortex to match the placement in real animal experiments (figure 7(A)). The peaks of *B*-field were underneath the two bases of the coil (figure 7(B)). The maximum strength was 460 mT on the surface of cortex. The sagittal view revealed that *B*-field decayed rapidly as the distance increases. It reduced to 100 mT at ∼3.8 mm from the surface. Simulated *E*-field distribution in the brain indicated that the focal point was under the center of the TMS coil (figure 7(C)). The maximum strength of *E*-field was estimated to be 7.2 V m^−1^ on the surface of cortex. The coil was able to generate an approximately 6-by-8 mm rectangular area with *E*-field strength above 3.6 V m^−1^ when it was tilted 15°. It could focally deliver TMS to the M1, secondary motor cortex (M2), and S1. Similar to *B*-field, the induced *E*-field decayed rapidly as the distance increased. It reduced to 1 V m^−1^ at ∼4.4 mm from the surface.

### 3.4. rTMS increased firing rates of primary somatosensory and motor cortical neurons

**Figure 8.**
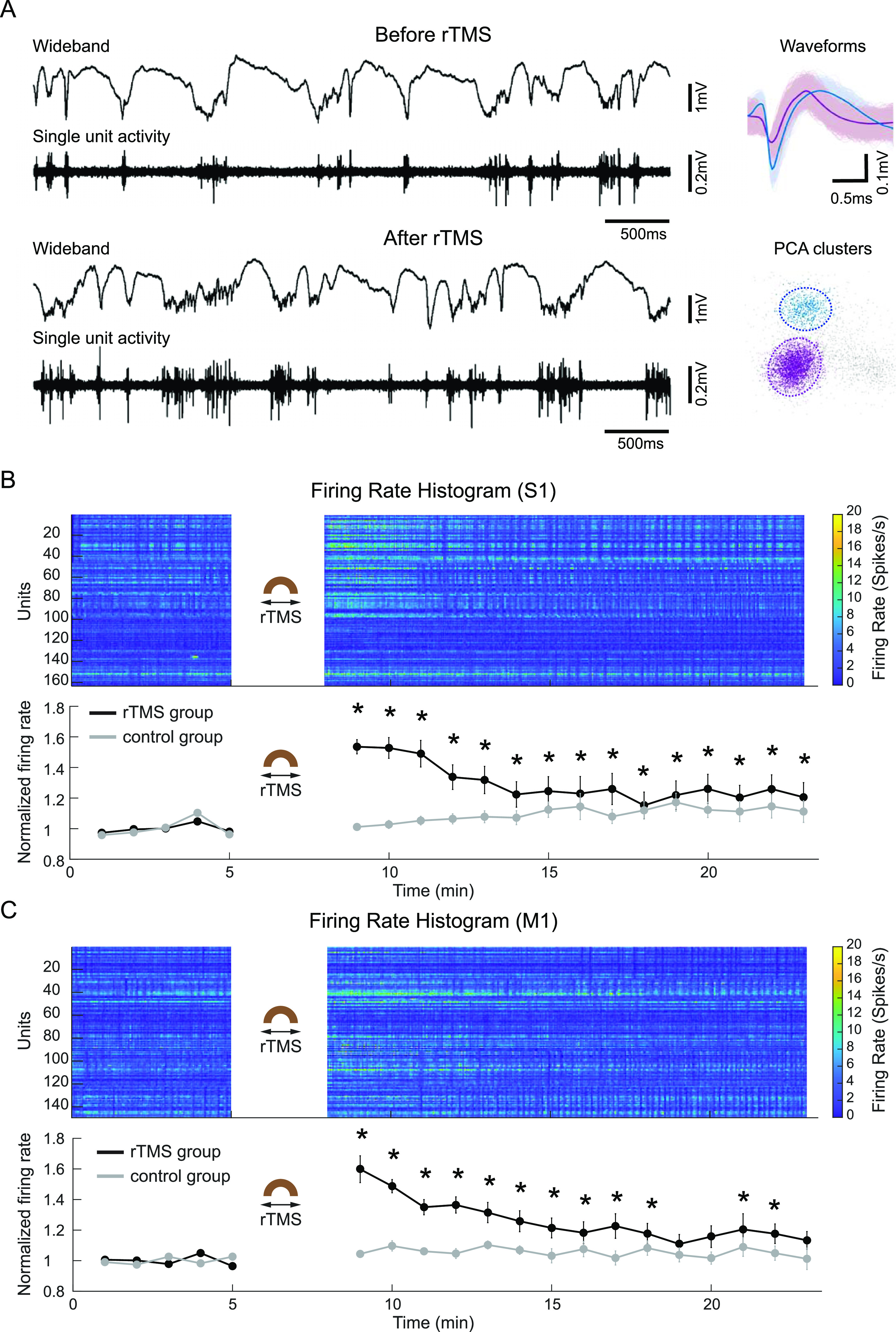
rTMS facilitated firings of S1 and M1 neurons. (A) Representative wideband and 250 Hz highpass filtered signals before and after rTMS, single-unit waveforms, and PCA clusters from one animal. (B) The firing pattern of S1 neurons before and after rTMS (*n* = 163) or control condition (*n* = 88). (C) The firing pattern of M1 neurons before and after rTMS (*n* = 149) or control condition (*n* = 101). In (B) and (C), top: firing rate histogram for rTMS group (bin size: 2 s); bottom: mean firing rate for both rTMS and control groups (bin size: 1 min). Comparisons of mean firing rates before and after rTMS or control condition were performed with two-tailed *t*-tests (*p < 0.01). Values were reported as the mean ± SEM.

To investigate the effect of subthreshold rTMS (3 min, 10 Hz) on cortical neurons, spontaneously occurring electrical activities from the S1 and M1 were recorded with the 64-channel multi-electrode array. Representative wideband and high-pass filtered (250 Hz) neural signals in one animal demonstrate the changes in single-unit recordings before and after rTMS (figure 8(A)). To further quantify the changes, spike sorting was performed to extract the SUA, and the firing rate of each neuron was calculated. Results showed that the firing rates of S1 (figure 8(B)) and M1 neurons (figure 8(C)) in ten animals (*n* = 5 per rTMS group) both increased after rTMS compared with the firing rates of baseline recordings. The normalized mean firing rates (S1 neurons: *n* = 163 per rTMS group, *n* = 88 per control group; M1 neurons: *n* = 149 per rTMS group, *n* = 101 per control group) were further averaged for every minute. The mean firing rates of S1 and M1 neurons significantly increased to 154 $ \pm $ 5% (*t* = 6.93, *p* = 0.002) and 160 $ \pm $ 9% (*t* = 7.20, *p* = 0.002) of the baseline level immediately after rTMS, respectively. They gradually decreased to 121 $ \pm $ 10% (*t* = 6.37, *p* = 0.003) and 113 $ \pm $6% (*p*> 0.01) over 15 min. Two-tailed *t*-test reveals no significant changes after control condition (*p*> 0.01).

### 3.5. rTMS suppressed SSEPs

**Figure 9.**
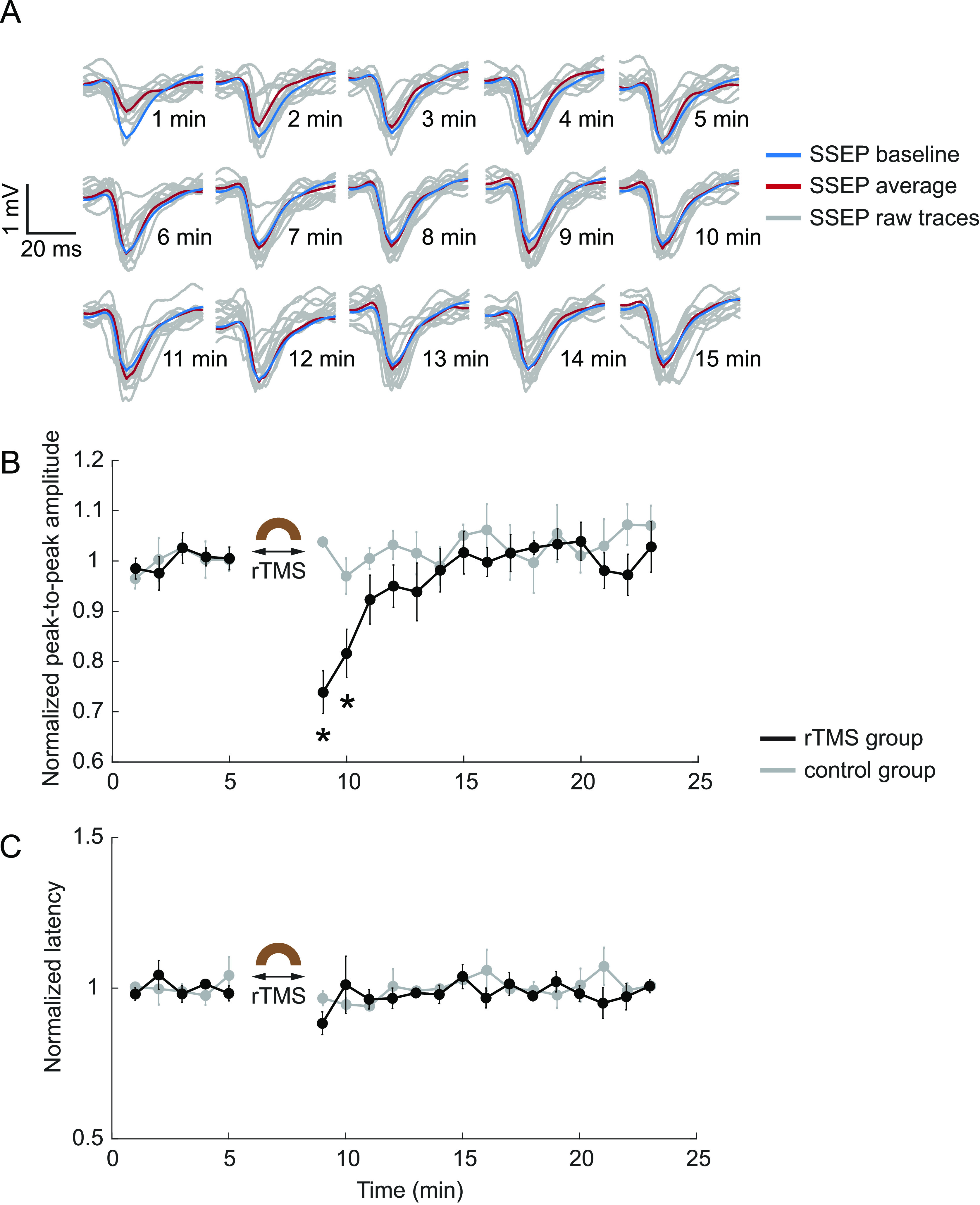
rTMS suppressed SSEPs. (A) Representative raw SSEP traces (gray) at 1–15 min after rTMS from one animal. Red: averaged trace; blue: baseline trace. (B) Mean SSEP amplitude before and after rTMS (*n* = 5) and control condition (*n* = 3). (C) Mean SSEP latency before and after rTMS (*n* = 5) and control condition (*n* = 3). In (B) and (C), comparisons of the amplitude or latency before and after rTMS or control condition were performed with two-tailed *t*-tests (*p < 0.01). Values were reported as the mean ± SEM.

To evaluate the effect of rTMS with the same stimulation parameters (3 min, 10 Hz) on the ascending sensory pathways, SSEPs elicited by electrical stimulation of the forelimb muscle were recorded from the contralateral S1 before and after rTMS. Representative traces of one animal demonstrate the suppression of SSEP following rTMS compared with the mean trace of baseline recordings (figure 9(A)). The normalized SSEP amplitude and latency of each group (*n* = 5 per rTMS group; *n* = 3 per control group) were further averaged for every minute. The SSEP amplitude was significantly suppressed to 74 $ \pm $ 4% (*t* = −11.9888*, p* = 0.0003) and 82 $ \pm $5% (*t*= −8.4367*, p*= 0.0011) of the baseline level during the first and second minute after rTMS, respectively (figure 9(B)). After ∼5 min, the SSEP amplitude returned to the baseline level. Two-tailed *t*-test reveals no significant changes after control condition (*p*> 0.01). The SSEP latency was obtained by calculating the duration between the stimulus artifact and the first negative peak of each evoked potential. Figure 9(C) demonstrates the average of normalized SSEP latency at each timepoint. It indicates that there is no significant change before and after rTMS and control condition (*p*> 0.01).

### 3.6. rTMS facilitated MEPs

**Figure 10.**
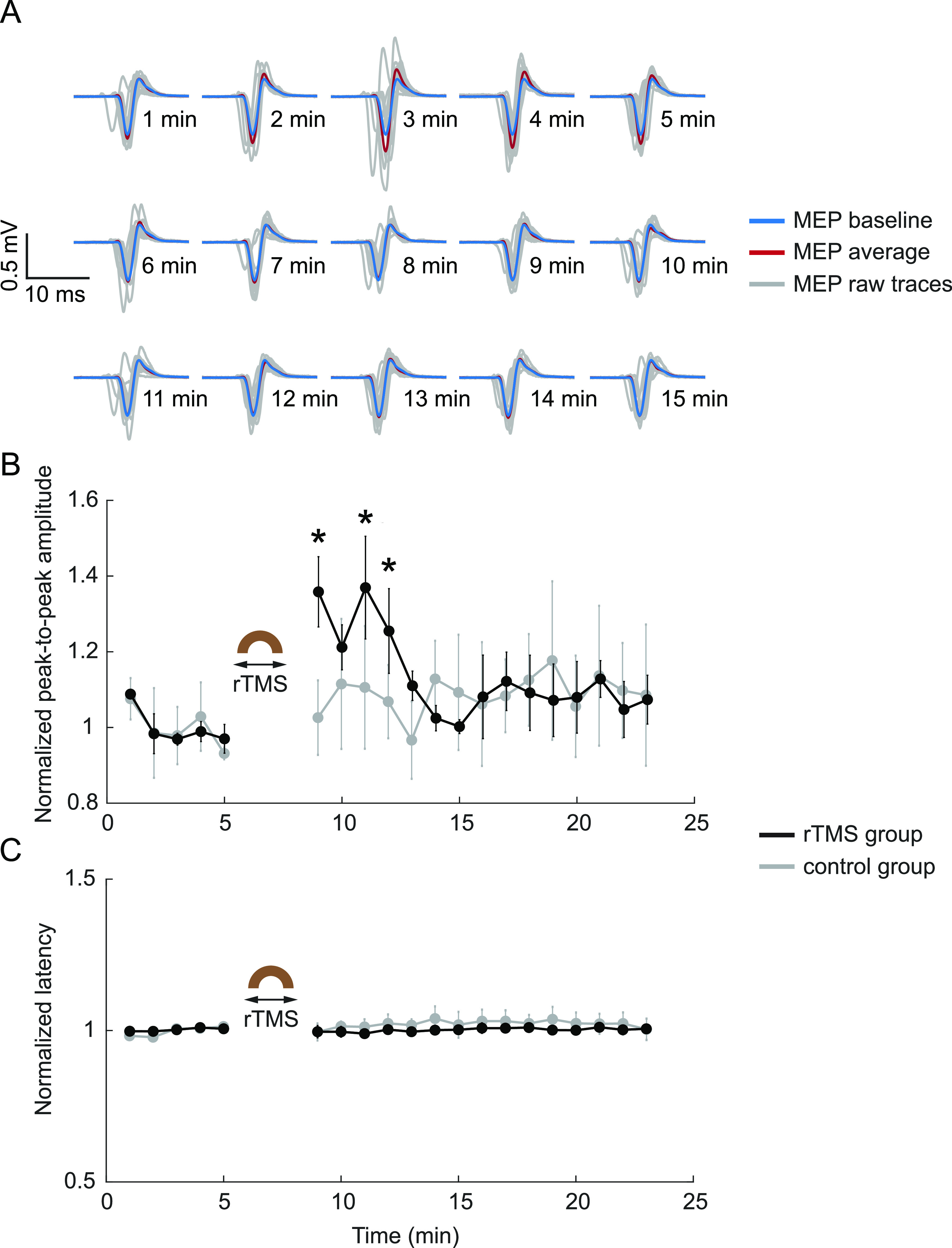
rTMS facilitated MEPs. (A) Representative raw MEP traces (gray) at 1–15 min after rTMS in a representative animal. Red: averaged trace; blue: baseline trace. (B) Mean MEP amplitude before and after rTMS (*n* = 5) and control condition (*n* = 3). (C) Mean MEP latency before and after rTMS (*n* = 5) and control condition (*n* = 3). In (B) and (C), comparisons of the amplitude or latency before and after rTMS or control condition were performed with two-tailed *t*-tests (*p < 0.01). Values were reported as the mean ± SEM.

To further compare the effects of rTMS (3 min, 10 Hz) on different pathways, MEPs representing the integrity and excitability of descending motor pathways were elicited by intracortical electrical stimulation of the M1 and recorded from the contralateral forelimb muscle before and after rTMS. Different from SSEP, representative traces demonstrate the facilitation of MEP following rTMS compared with the mean trace of baseline recordings (figure 10(A)). The normalized MEP amplitude and latency of each group (*n* = 5 per rTMS group; *n* = 3 per control group) were further averaged for every minute. The MEP amplitude significantly increased to 136 $ \pm $ 9% (*t* = 6.5420, *p* = 0.0028), 137 $ \pm $ 14% (*t* = 6.7441, *p* = 0.0025), and 126 $ \pm $11% (*t*= 4.6522*, p*= 0.0096) of the baseline level during the first, third, and forth minute after rTMS, respectively (figure 10(B)). After ∼5 min, the MEP amplitude returned to the baseline level. Two-tailed *t*-test reveals no significant changes after control condition (*p*> 0.01). The MEP latency was obtained by calculating the duration between the stimulus artifact and the first negative peak of each evoked potential. Figure 10(C) demonstrates the average of normalized MEP latency at each timepoint. Similar to SSEPs, there is no significant change in MEP latency before and after rTMS and control condition (*p*> 0.01).

## 4. Discussion

In this study, we developed and tested a novel miniaturized coil and its driving circuit for rodent TMS studies. As we knew, different TMS coil geometries resulted in different *B*- and *E*-field distributions, and further led to distinguishing effects of TMS on neural activities and behaviors. Our coil was designed with the main aims to (1) scale down the geometric size to generate focal *B*- and *E*-fields similar to those of human TMS coils, and (2) enable concurrent electrophysiological recording and stimulation at its focal point. The resulting design consisted of a C-shaped core with two adjacent but separate coil windings at the two bases, which carried currents in opposite directions. Like the conventional figure-eight coils, the opposite currents induced a focal *E*-field under the center of the coil. Different from the figure-eight coils, there was a gap between the two bases of the coil, which allowed convenient placements of electrodes.

Our coil had multiple advantages over existing ones. First, the use of magnetic core reduced the flux leakage and resulted in a more focal *B*-field [51]. In addition, the iron powder core was composed of ferromagnetic particles which were coated with organic compounds to ensure electrical insulation. It eliminated the eddy current in the core and largely reduced heat loss [37]. Although its permeability was lower than that of iron or ferrite core, it had higher saturation flux density [21, 38], which produced a significantly higher *B*-field intensity (473 mT measured at ∼0.5 mm) compared to most of the existing small coils [17–20, 24]. Second, our TMS coil contained less turns (30 turns) of copper wires. Higher winding density would lead to higher coil inductance, which in turn reduced the current in the coil [20]. Moreover, it would cause undesired heating which might result in irreversible damage on the coil as well as the biological tissue [52]. Third, as we stated above, our coil could be used in conjunction with standard electrophysiological recording and stimulation due to the air gap between its bases. The 5 mm gap allowed any rotation or placement of the multi-electrode array (1.4 mm width at the tip and 3.3 mm width at the end) used in this study. Additionally, a 150° angle between two bases of the coil fitted the curvature of the rat head and made the coil close to the brain surface. The coil was able to generate a focal *E*-field at the center of the base and at the same time allowed implantation of electrodes at the same location. Although the resulting *E*-field intensity (5.2 V m^−1^ measured at ∼3.5 mm in saline) was much smaller compared to those of human TMS coils (of the order of 100 V m^−1^ [27, 53]), it was higher than many of the existing miniaturized circular [17, 18, 20] and figure-eight [24] coils customized for rodents. Fourth, our TMS coil was able to deliver arbitrary stimulation pulse patterns with different intervals and amplitudes. These features are essential for optimizing stimulation parameters and further building a closed-loop TMS system to achieve precise neuromodulation.

We saw this combination of TMS, electrophysiological recording, and electrical stimulation in small animal brain as the most important feature of this miniaturized C-shape coil. As we knew, SUAs provided the most precise information of neuronal activities at the single-neuron level. The effects of rTMS on SUAs were highly informative about its effects on neural circuits, brain functions, and behaviors. In this study, we demonstrated that brief subthreshold rTMS could significantly facilitate the firings of S1 and M1 neurons. Previous studies showed that rTMS at 10 Hz decreased the GABAergic synaptic strength [54] and increased glutamatergic synaptic strength [55, 56] *in vitro*. These two forms of modifications of synaptic strengths might account for the increasing firing rates observed after rTMS in this study.

MEP elicited by electrical stimulation or single-pulse TMS over the M1 was one of the hallmark measures for the quality and integrity of the descending motor pathways [57, 58]. It was used to assess the effects of rTMS on the corticospinal tract excitability in both human and animal studies [19, 59–63]. In this work, we used MEPs elicited by electrical stimulation of layer 5 neurons in the M1 to evaluate the effects of subthreshold rTMS generated by our coil. Results showed that MEP amplitude significantly increased after rTMS. This increase of MEP amplitude was consistent with previous studies [19, 63]. The change of MEP amplitude provided indirect evidence on the neuronal mechanism of rTMS. It was likely that these changes were due to a mixture of changes on the intrinsic excitability of neurons and synaptic connectivity in the activated pathways [64, 65]. For example, the effects of rTMS on SUAs might contribute to its effects on MEPs; synaptic strengths might be modified between these neurons. In addition, MEP latency was another indicator of the corticospinal tract excitability, which reflected the neural conduction rate from the descending impulse to the target muscle [66]. Our result showed that the change in MEP latency after rTMS was less prominent.

In general, high frequency ($ &gt; $5 Hz) rTMS had a facilitatory effect on cortical excitability lasting for seconds to minutes, whereas low frequency ($ \leqslant $1 Hz) rTMS typically had an inhibitory effect [65, 67]. Interestingly, we observed a facilitatory effect of rTMS on SUAs and MEPs but an inhibitory effect on SSEPs with the same rTMS parameters (3 min, 10 Hz). The SSEP was elicited by electrical stimulation over the median nerve and recorded form the corresponding somatosensory cortex. It reflected the function and integrity of ascending sensory pathways [68, 69]. The SSEP was often used to evaluate the effects of rTMS in patients with migraine [70–72] or stroke [73, 74]. Our results showed that SSEP amplitude significantly decreased after rTMS. Meanwhile, SSEP latency, which represented the neural conduction rate from the ascending impulse to the target cortex, had less prominent changes after rTMS. Previous studies showed improvement in habituation of SSEP in migraineurs following 10 Hz rTMS treatment [70–72]. It suggested that the increase of plasma beta-endorphin level [72, 75] and the excitation of GABAergic neurons [70, 72] might account for the changes in sensitization and habituation. However, the inhibitory effect of rTMS on SSEP amplitude in healthy anesthetized subjects has not been studied yet. The neurobiological mechanisms of the effect of rTMS on SSEP require further investigations.

One main challenge of designing miniaturized coils was caused by the trade-off between coil diameter and *E*-field strength [76]. To make the coil size suitable for rodent, extremely high current was required to achieve the same stimulation strength as in human TMS coil [21, 34], which might cause excessive heating, electromagnetic stress, and other technical difficulties. Given this physical constraint, further improvements could still be made to the current design. For example, the size of the gap largely influenced the magnetic flux in a gapped toroid [77]. Optimization of coil performance could be made via a finite element model. We could further improve the focality of the *E*-field by optimizing the distance and angle of the gap of the C-shaped coil. In addition, our current DC voltage source had a maximal output of 60 V and might not provide sufficient power for higher rTMS frequency to prevent the drop of the pulse amplitude. We could modify the driving circuit and use a more powerful DC power supply to increase the coil current. Furthermore, it was noted that the temperature of the coil increased ∼23° after rTMS (3 min, 10 Hz). Since the resistance of the coil remained low (0.1 Ω) at the frequency of the input pulse, the rise of temperature was likely caused by the core losses. The electrical properties of the coil could change due to the increased temperature of the windings, which might partly explain the drop of coil current at higher rTMS frequency. A temperature control or cooling system might be required when the current is further increased, since heating of the coil would potentially lead to a local brain temperature change that might influence neural excitability [78–80]. Moreover, the coil generated light vibrations and sounds during stimulation. The sound could potentially affect the auditory sensory pathways [81, 82]. However, the ears of the animal were protected and plugged with Parafilm-wrapped ear bars throughout the experiments under anesthesia, hence the sound should have negligible effects on the auditory sensory system. It was worth noting that the control condition (rTMS turned off) in this study was different from the sham condition for conventional figure-eight coils. In sham condition, the current of the two circular windings flowed in the same direction to cancel the *E*-field right under the center of the coil. Hence, sham TMS could reproduce the vibration, sound, and superficial *E*-field on the head but not cause direct neural effects in the cortex as the active TMS [83–86]. To realize an appropriate sham condition for our coil, modifications could be made by dividing the 30 windings into two groups of 15 windings. Each group of windings could be driven by the circuit separating from the parallel system with opposite current directions. Besides, Gaussian pulses were used as the input signal to the stimulator. Different from the conventional monophasic pulse, the Gaussian pulse had a more symmetric waveform similar to the conventional half-sine pulse [87]. The effect of TMS with different TMS parameters, including pulse shape, frequency, duration, and intensity, remained to be further studied.

It is widely accepted that the neurobiological effects of TMS are mediated through the *E*-field induced in the brain [2, 88, 89]. The induced *E*-field is not only determined by the TMS coil, but also influenced by the geometry and conductivity of the head [27, 90]. In this study, due to a large craniotomy performed on the rat head, a brain-only model was used to simulate the *E*-field distribution for simplicity. The resulting *E*-field was less accurate than a full head model, since tissue boundaries with a contrast in conductivity would cause the accumulation of surface charges whose secondary *E*-field significantly affected the primary *E*-field [91]. Further improvements of the *E*-field simulation could be made using a realistic head model with all experimental conditions. To be noted, the main purpose of the *E*- and *B*-field measurements in this study was to compare with the simulated results under identical conditions to validate the finite element model. The measured *E*-field in saline differed from the *E*-field induced in free space or the brain due to the secondary *E*-field created by the accumulation of surface charges at the boundaries of the beaker. Besides, the glass of the beaker prevented measurement of *E*-field closer to the coil. To have a more accurate measurement of the induced *E*-field, a saline-free method [92] would be considered for future experiments.

In summary, we designed, fabricated, and validated a novel miniaturized C-shaped TMS coil and tested it in rat experiments. We reported the methodology of developing and characterizing the miniaturized TMS coil and its stimulator design, experimental measurement, and simulation of field distributions. The efficacy of this coil in neuromodulation was validated with electrophysiological recordings of SUAs, SSEPs, and MEPs in rats. This miniaturized coil and the associated experimental paradigm enabled the combination of TMS, electrophysiological recording, and electrical stimulation in rat brains. It provided a powerful tool to investigate the neural responses and underlying mechanisms of TMS in small animal models. Using this paradigm, we for the first time observed distinct modulatory effects on SUAs, SSEPs, and MEPs with the same rTMS protocol in anesthetized rats. These results suggested that multiple neurobiological mechanisms in the sensorimotor pathways were differentially modulated by rTMS. In the future, we will extend these studies to awake and behaving animals to further investigate the neurobiological mechanisms of TMS and make the connections between electrophysiological activities to behaviors and cognitive functions.

## Data Availability

All data that support the findings of this study are included within the article (and any supplementary files).
